# The host response in different aetiologies of community-acquired pneumonia

**DOI:** 10.1016/j.ebiom.2022.104082

**Published:** 2022-06-01

**Authors:** Alex R. Schuurman, Tom D.Y. Reijnders, Tjitske S.R. van Engelen, Valentine Léopold, Justin de Brabander, Christine van Linge, Michiel Schinkel, Liza Pereverzeva, Bastiaan W. Haak, Xanthe Brands, Maadrika M.N.P. Kanglie, Inge A.H. van den Berk, Renée A. Douma, Daniël R. Faber, Prabath W.B. Nanayakkara, Jaap Stoker, Jan M. Prins, Brendon P. Scicluna, W. Joost Wiersinga, Tom van der Poll

**Affiliations:** a Centre for Experimental and Molecular Medicine (CEMM), Amsterdam University Medical Centres - Location AMC, University of Amsterdam, Amsterdam, the Netherlands; b Department of Anaesthesiology and Intensive Care, GH St Louis-Lariboisière, Inserm UMR-S 942 (MASCOT), Université de Paris, 75010 Paris, France; c Department of Radiology, Amsterdam University Medical Centres, University of Amsterdam, Amsterdam, The Netherlands; d Department of Internal Medicine, Flevo Hospital, Almere, the Netherlands; e Department of Internal Medicine, BovenIJ Hospital, Amsterdam, the Netherlands; f Department of Internal Medicine, Amsterdam University Medical Centres, University of Amsterdam, Amsterdam, the Netherlands; g Centre for Molecular Medicine and Biobanking, University of Malta, Malta; h Department of Applied Biomedical Science, Faculty of Health Sciences, Mater Dei hospital, University of Malta, Malta

**Keywords:** Community-acquired pneumonia, COVID-19, Streptococcus pneumoniae, Influenza, Host response, Aetiology

## Abstract

**Background:**

Community-acquired pneumonia (CAP) can be caused by a variety of pathogens, of which *Streptococcus pneumoniae*, Influenza and currently SARS-CoV-2 are the most common. We sought to identify shared and pathogen-specific host response features by directly comparing different aetiologies of CAP.

**Methods:**

We measured 72 plasma biomarkers in a cohort of 265 patients hospitalized for CAP, all sampled within 48 hours of admission, and 28 age-and sex matched non-infectious controls. We stratified the biomarkers into several pathophysiological domains- antiviral response, vascular response and function, coagulation, systemic inflammation, and immune checkpoint markers. We directly compared CAP caused by SARS-CoV-2 (COVID-19, n=39), *Streptococcus pneumoniae* (CAP-strep, n=27), Influenza (CAP-flu, n=22) and other or unknown pathogens (CAP-other, n=177). We adjusted the comparisons for age, sex and disease severity scores.

**Findings:**

Biomarkers reflective of a stronger cell-mediated antiviral response clearly separated COVID-19 from other CAPs (most notably granzyme B). Biomarkers reflecting activation and function of the vasculature showed endothelial barrier integrity was least affected in COVID-19, while glycocalyx degradation and angiogenesis were enhanced relative to other CAPs. Notably, markers of coagulation activation, including D-dimer, were not different between the CAP groups. Ferritin was most increased in COVID-19, while other systemic inflammation biomarkers such as IL-6 and procalcitonin were highest in CAP-strep. Immune checkpoint markers showed distinctive patterns in viral and non-viral CAP, with highly elevated levels of Galectin-9 in COVID-19.

**Interpretation:**

Our investigation provides insight into shared and distinct pathophysiological mechanisms in different aetiologies of CAP, which may help guide new pathogen-specific therapeutic strategies.

**Funding:**

This study was financially supported by the Dutch Research Council, the European Commission and the Netherlands Organization for Health Research and Development.

**Research in context**

**Evidence before this study**

We searched PubMed for research articles from January 1 2020 to November 1, 2021. Hundreds of observational studies were published describing the host response in COVID-19. Only a few of these studies, mostly restricted to patients on the intensive care unit, compared patients with COVID-19 to other aetiologies of community-acquired pneumonia (CAP). These studies mainly reported on a specific part of the host response, such as enhanced cell-mediated antiviral responses in severe COVID-19.

**Added value of this study**

In this large multicentre cohort of 265 patients hospitalized for CAP, a set of 72 plasma biomarkers was measured to obtain information on five pathophysiological domains: cell-mediated antiviral response, activation and function of the vasculature, systemic inflammation, coagulation and immune checkpoint markers. This study directly compares these markers and domains between patients with different causative pathogens of CAP (SARS-CoV-2, *Streptococcus pneumonia,* and Influenza), in an analysis that was adjusted for age, sex and disease severity. This investigation separates pathogen-specific immune features from the common host response to infection, which improves our knowledge of CAP pathophysiology across different aetiologies.

**Implications of all the available evidence**

A better understanding of the host response in the wider population of patients with CAP can help generate new hypotheses, and guide therapeutic strategies tailored to specific causative pathogens. For example, several trials in COVID-19 have been performed with the aim of limiting endothelial dysfunction. As the data in this study suggest that the endothelial barrier integrity is more disturbed in non-COVID-19 CAP, it may be interesting to expand this rationale to CAP aetiologies other than COVID-19.

Alt-text: Unlabelled box

## Introduction

The global burden of pneumonia is vast: an estimated 450 million people are affected by lower respiratory tract infections yearly, resulting in 6.8 million hospitalizations for pneumonia and 1.1 million in-hospital deaths.^1^^,^^2^ Community-acquired pneumonia (CAP) can be caused by many different pathogens, but the causative agent remains unidentified in approximately half of hospitalized patients. A small group of microbes, including S*treptococcus pneumoniae* and Influenza viruses, are responsible for the majority of cases in which a pathogen can be identified.^3^ Since late 2019, the microbial aetiology of CAP has shifted dramatically with the emergence of SARS-CoV-2, the virus that causes COVID-19. More than 350 million infections and 5 million deaths related to COVID-19 have been reported, whereas the incidence of non-COVID-19 pneumonia strongly decreased, in part due to preventative societal measures.^4^^,^^5^

Although COVID-19 pneumonia shares many features with CAP caused by other pathogens, some characteristics are highly distinctive. COVID-19 has a fairly idiosyncratic disease course and leads to more severe disease in patients with metabolic comorbidities such as obesity and diabetes mellitus type 2.^6^ Dysregulation of the host response is a pivotal pathophysiological feature in all forms of CAP, and many of the reported host response characteristics of COVID-19-including systemic inflammation, endothelial cell activation and altered immune cell activation^7^ are also well-established features of non-COVID-19 CAP.^2^^,^^7^ Despite these ostensible similarities, studies directly comparing COVID-19 with other aetiologies of CAP are scarce. Direct comparisons may help separate pathogen-specific immune features from the common characteristics of a dysregulated host response during pneumonia. This may improve our understanding of CAP pathophysiology across different aetiologies, and thereby inform therapeutic strategies tailored to specific pathogens in CAP.

Here, we measured an extensive set of plasma biomarkers indicative of key pathophysiological domains in patients hospitalized due to CAP-representative of the patient population prior to the SARS-CoV-2 pandemic-or COVID-19, and delineate common and unique host response features of different aetiologies of CAP.

## Methods

### Patients

Patients were recruited as part of the ELDER-BIOME study (clinicaltrials.gov identifier NCT02928367) or OPTIMACT study (Dutch Trail Register identifier NTR6163), both approved by the medical ethical committee of the Amsterdam University Medical Centers. Other participating sites included the BovenIJ Hospital, Flevo Hospital and the Spaarne Gasthuis Hospital. Written informed consent was obtained from all participants or their legal representatives. Trained research physicians screened patients older than 18 years admitted between October 2016 and June 2018 (for the OPTIMACT) or June 2020 (for the ELDER-BIOME) for eligibility. All patients with COVID-19 were included in April 2020 and May 2020, before the SARS-CoV-2 Alpha variant became dominant in the Netherlands.

Patients were eligible if they were admitted to the hospital and met all of the following criteria: clinical suspicion of an acute infection of the respiratory tract, defined as the presence of at least one respiratory symptom (new cough or sputum production, chest pain, dyspnea, tachypnea, abnormal lung examination, or respiratory failure) and one systemic symptom (documented fever or hypothermia, leukocytosis or leukopenia), and an evident new or progressive infiltrate, consolidation or pleural effusion on chest X-ray or computed tomography scan. Patients were excluded if there was a clinical suspicion of aspiration pneumonia or hospital-acquired pneumonia.

All baseline and clinical variables were scored from the electronic health records. Severity scores - the Pneumonia Severity Index (PSI), the Modified Early Warning Score (MEWS), the CURB-65 and the quick Sequential Organ Failure Assessment qSOFA score-were calculated upon hospital admission. An overview of missing data in clinical variables, which can be considered missing at random, is shown in Supplemental Table I. Ethylenediaminetetraacetic acid (EDTA) anticoagulated blood was obtained within 48 hours of hospital admission (within 24 hours for 246/265 [92·8%] patients). Several COVID-19 patients received immunomodulatory drugs as part of clinical trials, but all samples were obtained prior to the first administration of these drugs. No COVID-19 patients were treated with dexamethasone prior to sampling (not standard of care during the period of enrolment). Age and sex-matched subjects recruited from the outpatient clinics without signs of an infection were included as controls.

### (Primary) pathogen assessment

We compared patients based on the aetiology of CAP: non-COVID-19 CAP (all patients without COVID-19), CAP-strep (primary pathogen *Streptococcus pneumoniae)*, CAP-flu (primary pathogen Influenza A or B), CAP-other (non-COVID-19 CAP excluding CAP-strep and CAP-flu), and COVID-19 (primary pathogen SARS-CoV-2). A team of research physicians assessed the clinical microbiology results obtained around time of admission, in combination with the clinical notes in the electronic health records. If an antigen urine test was positive for *Streptococcus pneumoniae,* we considered this proof of infection. If both *Streptococcus pneumoniae* and Influenza were identified (in three patients), we considered the bacterium the primary pathogen. 37/39 patients with COVID-19 had reverse transcription (RT-PCR)-confirmed SARS-CoV-2 infection, and a clinical diagnosis was made in the other two patients with COVID-19 based on case history and radiological findings.

### Assays

We measured host response biomarkers using Luminex (R&D, USA) and cytometric bead array (CBA; BioLegend, USA). Details are provided in Supplemental Table 2. For both Luminex and CBA, measurements below the limit of quantification were imputed as half the lower limit of quantification. Luminex measurements above the upper limit of quantification (eight datapoints) were set to the upper limit of quantification. CBA measurements above the upper limit of quantification did not occur.

### Statistics

All tests were two-sided and a P-value<0·05 (corrected for multiple testing where mentioned) was considered statistically significant. All biomarker data were transformed to a normal distribution using the Box-Cox method prior to statistical analysis.^8^ For the volcano plots, biomarker levels were compared using Welch's *t*-test, corrected for multiple testing using the Benjamini-Hochberg (BH) method. To account for unequal variances, we performed a White-adjusted ANOVA to test overall differences between groups, if significant followed by post-hoc pairwise Games-Howell tests. We performed a White-adjusted ANCOVA including covariates related to biomarkers and/or the pathogen group: age, sex, and disease severity scores [PSI, CURB-65, MEWS^9^ and qSOFA]. We opted to restrict these models to variables known to be strongly related to the acute phase of the host response to infection and which had sufficient data in each subgroup. We used a listwise deletion approach for the ANCOVA: 17 out of 265 patients were excluded due to missing data in one or more covariates (Table S1; fraction of missing information 6·4%). For the effect size heatmaps, we calculated Hedges’ *g*^10^ between patient groups and control subjects. Biomarker data were scaled prior to Principal Component Analysis (PCA). Differences on the PCA plots were tested by ANOVA and post-hoc pairwise Games-Howell tests of the first and second principal component. We performed several subgroup and sensitivity analyses, as specified in the final paragraph of the Results section. All statistical analyses were performed using R version 4.0.4 (Vienna, Austria).

### Ethics

The study was approved by the medical ethical committee of the Amsterdam University Medical Centers (NL57923.018.16 for OPTIMACT, NL57847.018.16 for ELDERBIOME). Written informed consent was obtained from all participants or their legal representatives.

### Role of funders

Funders did not have any role in study design, data collection, data analyses, interpretation, or writing of report.

## Results

### Patient characteristics and clinical outcomes

We analysed 265 consecutively enrolled patients hospitalized for CAP. SARS-CoV-2 was designated the primary pathogen in 39 patients (COVID-19), *Streptococcus pneumoniae* in 27 patients (CAP-strep), Influenza in 22 patients (CAP-flu; 16 Influenza A, 6 Influenza B); and 177 patients had CAP of other or unknown aetiology (CAP-other). Baseline characteristics and outcomes of all patient groups are shown in Table 1 (Supplemental Table 3 shows all patients versus 28 age- and sex-matched non-infectious control subjects). Patients with COVID-19 were slightly younger (non-significant), had a higher body mass index (BMI), and a longer duration of symptoms prior to hospital admission, in line with earlier reports.^11^ Leukocyte and neutrophil numbers were higher in patients with CAP-other and CAP-strep, and relatively low in COVID-19. Admission disease severity scores reflecting changes in vital signs were comparable between groups (MEWS and qSOFA), but disease severity scores including age and/or comorbidities (PSI and CURB-65) were somewhat higher in patients with non-COVID-19 CAP. Clinical outcomes were similar between patient groups.

**Table 1:** Clinical characteristics and disease course of patients.

	CAP-other*	CAP-strep	CAP-flu	COVID-19	p-value
	(n = 177)	(n = 27)	(n = 22)	(n = 39)	
**DEMOGRAPHICS**					
Age, years	67·8 (16·6)	64·4 (13·3)	65·8 (15·8)	60·5 (11·4)	0·06
Sex, male	99 (55·9)	17 (63·0)	12 (54·5)	20 (51·3)	0·82
Body mass index	25·8 (5·5)	24·2 (5·5)	26·1 (5·4)	30·3 (6·9)↑	<0·01
**CHRONIC COMORBIDITIES**					
Immune deficiency	36 (20·3)	4 (14·8)	2 (9·1)	2 (5·1)	0·09
Immunosuppressives	29	2	1	2	
Solid organ transplant	19	0	2	2	
Haematological malignancy	12	1	1	0	
Chemotherapy <6 months	6	0	0	0	
COPD	52 (29·4)	10 (37·0)	7 (31·8)	2 (5·1)↓	<0·01
Asthma	15 (8·5)	3 (11·1)	2 (9·1)	3 (7·7)	0·92
Congestive heart failure	17 (9·6)	2 (7·4)	0 (0·0)	1 (2·6)	0·31
Myocardial infarction	30 (16·9)	4 (14·8)	3 (13·6)	2 (5·1)	0·29
History of stroke	21 (11·9)	0 (0·0)	0 (0·0)	3 (7·7)	0·09
Diabetes mellitus	49 (27·7)	6 (22·2)	3 (13·6)	10 (25·6)	0·56
Chronic kidney disease	23 (13·0)	1 (3·7)	2 (9·1)	2 (5·1)	0·37
**LABORATORY TESTS**†					
Leukocytes, x10^9^/L	11·8 [8·9, 14·7]↑	13·7 [8·8, 19·2]	9·9 [5·3, 13·8]	6·9 [5·7, 9·0]↓	<0·01
Neutrophils, x10^9^/L	9·1 [6·1, 12·2]↑	12·2 [8·9, 16·3]↑	8·8 [4·8, 11·2]	5·1 [3·9, 6·9]↓	<0·01
Lymphocytes, x10^9^/L	0·94 [0·62, 1·52]	0·86 [0·60, 1·20]	1·08 [0·56, 1·23]	0·97 [0·72, 1·40]	0·93
Platelets, x10^9^/L	252 (117)	243 (127)	206 (96)	261 (108)	0·31
Creatinine, µmol/L	89 [66, 124]	88 [72, 115]	85 [70, 110]	88 [71, 102]	0·90
**VITAL SIGNS AND DISEASE EVERITY**†					
Temperature,°C	38·2 (1·2)	38·4 (1·0)	38·1 (1·0)	37·7 (1·2)↓	0·03
Respiratory rate, bpm	22 [16, 26]	22 [19, 30]	23 [20, 25]	23 [20, 27]	0·12
Heart rate, bpm	98 [84, 110]	103 [91, 121]	90 [80, 100]	94 [80, 108]	0·03
MAP, mmHg	97·3 (16·8)	88·3 (13·8)	91·2 (18·8)	96·4 (15·4)	0·03
MEWS	3 [2, 4]	4 [2, 5]	3 [3, 4]	3 [2, 4]	0·14
PSI	4 [3, 4]	4 [3, 4]	4 [2, 4]	3 [2, 3]↓	<0·01
CURB-65	2 [1, 2]	2 [1, 3]	1 [0, 3]	1 [0, 2]↓	0·01
qSOFA	1 [0, 1]	1 [0, 1]	0 [0, 1]	1 [0, 1]	0·21
**DISEASE COURSE**					
Symptoms to admission, days	3 [2, 7]↓^,^‡	4 [3, 7]‡	4 [2, 6]‡	8 [5, 10]↑	<0·01
Increase of corticosteroids upon admission	28 (15·8)	5 (18·5)	7 (31·8)	2 (5·1)	0.05
ICU admission (at any point during admission)	15 (8·5)	4 (14·8)	1 (4·5)	6 (15·4)	0·36
Time to clinical stability¶ or discharge, days	3 [2, 6]	3 [2, 6]	3 [2, 5]	4 [3, 7]	0·22
Hospital LOS, days	5 [3, 9]	4 [3, 9]	3 [2, 4]	4 [3, 8]	0·11
28-day mortality	11 (6·2)	0 (0·0)	1 (4·5)	5 (12·8)	0·20

CAP = community-acquired pneumonia; COPD = chronic obstructive pulmonary disease; bpm = breaths/beats per minute; MAP = mean arterial pressure; CURB-65 = confusion, blood urea nitrogen, respiratory rate, blood pressure, age 65 or older; MEWS= modified early warning score; PSI= pneumonia severity index; qSOFA = quick sequential organ failure assessment score; LOS= length of stay.

Continuous data are presented as mean (standard deviation) or median [interquartile range], and compared using a two-sided ANOVA or two-sided Kruskal-Wallis test, respectively. Categorical data are presented as count (percentage) and compared using Fisher's exact test.

⁎ Microbiological results in this group: no pathogen identified (n=120), *Haemophilus influenzae* (n=15), *Pseudomonas aeruginosa* (n=8), Rhinovirus (n=7), Human metapneumovirus (n=5), Coronavirus (n=4), *Staphylococcus aureus* (n=4), *Klebsiella pneumoniae* (n=2), Respiratory syncytial virus (n=2), *Pneumocystis jirovecii* (n=2), Parainfluenza virus 1-4 (n=2), Aspergillus spp. (n=1), Legionella (n=1), *Mycobacterium tuberculosis* (n=1), *Escherichia coli* (n=1), *Mycoplasma pneumoniae* (n=1), *Rothia dentocariosa* (n=1).

† Measured upon presentation to the emergency department

‡ Missing values in 50/177 (28·2%), 3/27 (11·1%), and 7/22 (31·8%) of patients; date of start of symptoms was not available in the OPTIMACT study

¶ Defined as the modified Halm's criteria: temperature ≤37·2°C, heart rate ≤100 bpm, systolic blood pressure ≤90 mmHg, respiratory rate ≤ 24 bpm, and oxygen saturation ≥90% for the entire day

↑ Significantly higher than grand mean of other groups, corrected for multiple testing by Holm's method.

↓ Significantly lower than grand mean of other groups, corrected for multiple testing by Holm's method.

### Distinctive and overlapping host response biomarker profiles in patients with non-COVID-19 CAP and COVID-19

**Figure 1:**
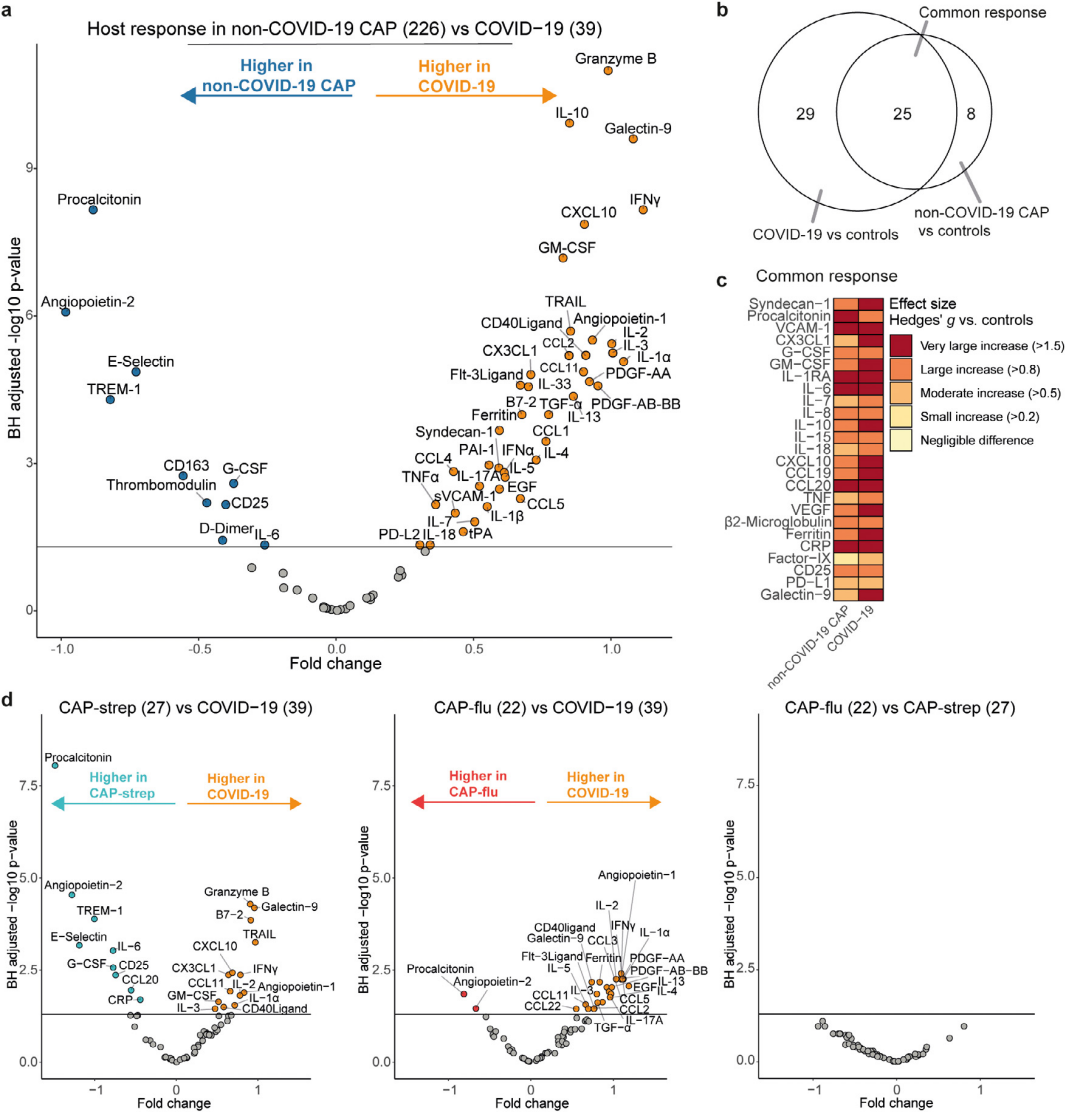
Common and distinct host response biomarkers in patients with community-acquired pneumonia with different microbial aetiologies. **a)** Volcano plot comparing plasma biomarkers between patients with non-COVID-19 CAP (n=226) and patients with COVID-19 (n=39). X-axis depicts the fold change of Box-Cox transformed values between groups, Y-axis depicts the Benjamini-Hochberg adjusted P-value. **b)** Venn-Euler plot showing the number of biomarkers that are significantly different from the non-infectious control group, either specific for COVID-19, specific for non-COVID-19 CAP or common between the two disease groups. **c)** Heatmap depicting the Hedges’ *g*, a measure of effect size, between the disease groups and controls for all biomarkers in the common response. **d)** Volcano plots showing plasma biomarkers for CAP-strep (n=27) versus COVID-19 (n=39), CAP-flu (n=22) versus COVID-19 (n=39), and CAP-flu (n=22) versus CAP-strep (n=27). X-axis depicts the fold change of Box-Cox transformed values between groups, Y-axis depicts the Benjamini-Hochberg adjusted p-value.

We analysed 72 host response biomarkers in all patients within 48 hours after admission (Supplemental Table 2). First, we compared the plasma concentrations of all biomarkers between patients with non-COVID-19 CAP and patients with COVID-19 (Supplemental Table 4). 50 biomarkers were significantly different between these two patient groups (BH-adjusted P<0·05; 10 higher in non-COVID-19 CAP, 40 higher in COVID-19; Figure 1a). The top differentially abundant biomarker in non-COVID-19 CAP was procalcitonin, an acute phase pro-peptide extensively investigated as a possible biomarker to diagnose bacterial infections.^12^ In COVID-19, the most significantly increased biomarker was granzyme B, an apoptosis-mediating serine protease that is mostly produced by natural killer cells and cytotoxic T cells, critical for eliminating virus-infected cells.^13^

To delineate common and unique host response features in non-COVID-19 CAP and COVID-19, we compared both disease groups to control subjects (Figure 1b). The common response profile-the biomarkers that were significantly different in both pneumonia groups relative to controls-consisted of 25 biomarkers, whereas the COVID-19 and non-COVID-19 CAP-specific responses comprised 29 and 8 markers, respectively (Supplemental Table 5). Figure 1c illustrates the biomarkers of the common response and depicts the effect size (Hedges’*g*) of the difference with control subjects.

### Direct comparison of the host response in community-acquired pneumonia caused by SARS-CoV-2, S. pneumoniae or Influenza A/B

We next investigated whether common pathogens in CAP associate with unique host response profiles. Directly comparing COVID-19 with CAP-strep revealed differences similar to the comparison with the overall CAP group (Figure 1d). Comparing COVID-19 with another viral aetiology, CAP-flu, resulted in differentially abundant biomarkers including various cytokines and chemokines (higher in COVID-19). Interestingly, we found no significant differences between CAP-strep and CAP-flu. Taken together, patients with COVID-19 displayed a distinct host response when compared with patients with non-COVID-19 CAP, although we also identified a substantial overlap in the host response between the different CAP aetiologies.

Next, we grouped the biomarkers into several pathophysiological domains-antiviral response, vascular response and function, coagulation, systemic inflammation, and immune checkpoint markers (Supplemental Table 2) - and compared their profiles in CAP-strep, CAP-flu, other non-COVID-19 CAP and COVID-19. Such a literature-based classification provides structure, and allows for a more holistic assessment of biomarkers that represent a specific aspect of the host response.^14^

### Antiviral cell-mediated response

**Figure 2:**
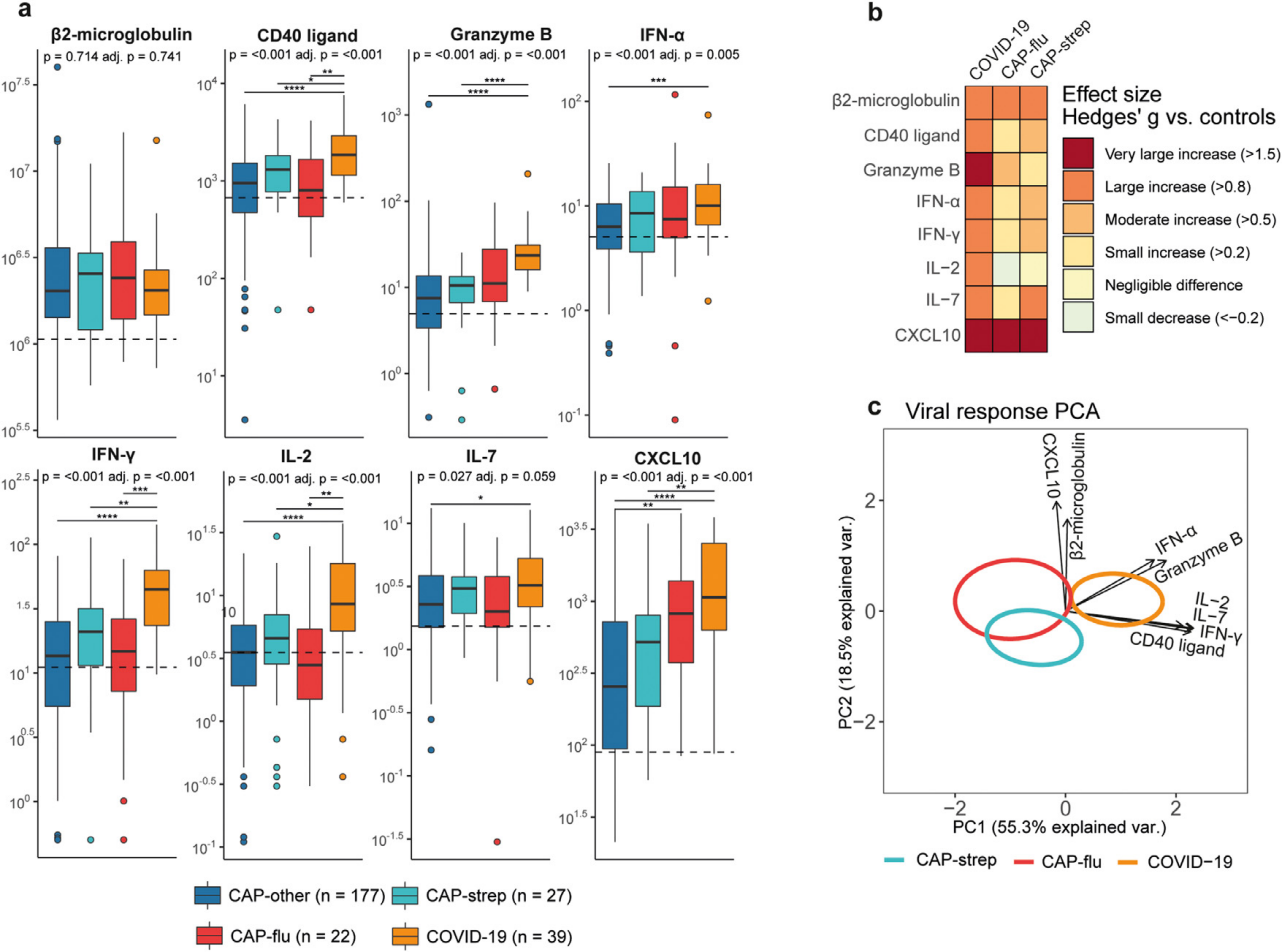
Biomarker levels reflective of cell-mediated antiviral responses in patients with community-acquired pneumonia with different microbial aetiologies. **a)** Boxplots comparing cell-mediated antiviral response markers between the CAP groups. The Y-axis depicts biomarker concentrations in pg/ml (prior to Box-Cox transformation). The median value for control subjects is shown as a dashed line. The p-value listed in each boxplot is obtained from the White-adjusted ANOVA, the adjusted (adj.) P-value was obtained from the White-adjusted ANCOVA model including age, sex, and disease severity scores as covariates. The stars depict the significance of the post-hoc pairwise Games-Howell tests performed after a significant ANOVA. *P<0·05, **P<0·01, ***P<0·001, ****P<0·0001. **b)** Heatmap depicting the Hedges’ *g* between the disease groups and controls for all antiviral response markers. **c**) Principal component analysis (PCA) of all viral response markers. X-axis label shows the percentage of explained variance on principal component 1, Y-axis label shows the percentage of explained variance on principal component 2. The ellipse indicates the central 10% of the groups. The arrows indicate the direction (arrow orientation) and strength (arrow length) of the correlation between each marker and the principal components.

Previous investigations reported on enhanced antiviral cell-mediated response in COVID-19.^15^ As a “proof of concept” that our approach would be able to detect differences in host response profiles reflective of certain pathophysiological domains in our cohort, we first compared biomarkers involved in the antiviral response between groups. Indeed, we found marked elevations of CD40 ligand, granzyme B, IFN-α, IFN-γ, IL-2, IL-7 and CXCL10, especially in COVID-19 (ANOVA for overall between group differences P<0·05 and Games-Howell for post-hoc pairwise comparisons P<0·05, Figure 2a+b). Figure 2c combines the preceding univariate analyses into in a multivariate analysis, and depicts a principal component analysis (PCA) plot showing the pathogen-specific disease groups (CAP-strep, CAP-flu and COVID-19) based on all antiviral response biomarkers. The PCA plot showed COVID-19 patients were clearly separated from the other groups (ANOVA P<0·001 and Games-Howell P<0·01 for PC1 scores between COVID-19 and both other groups).

### Vascular responses and function

**Figure 3:**
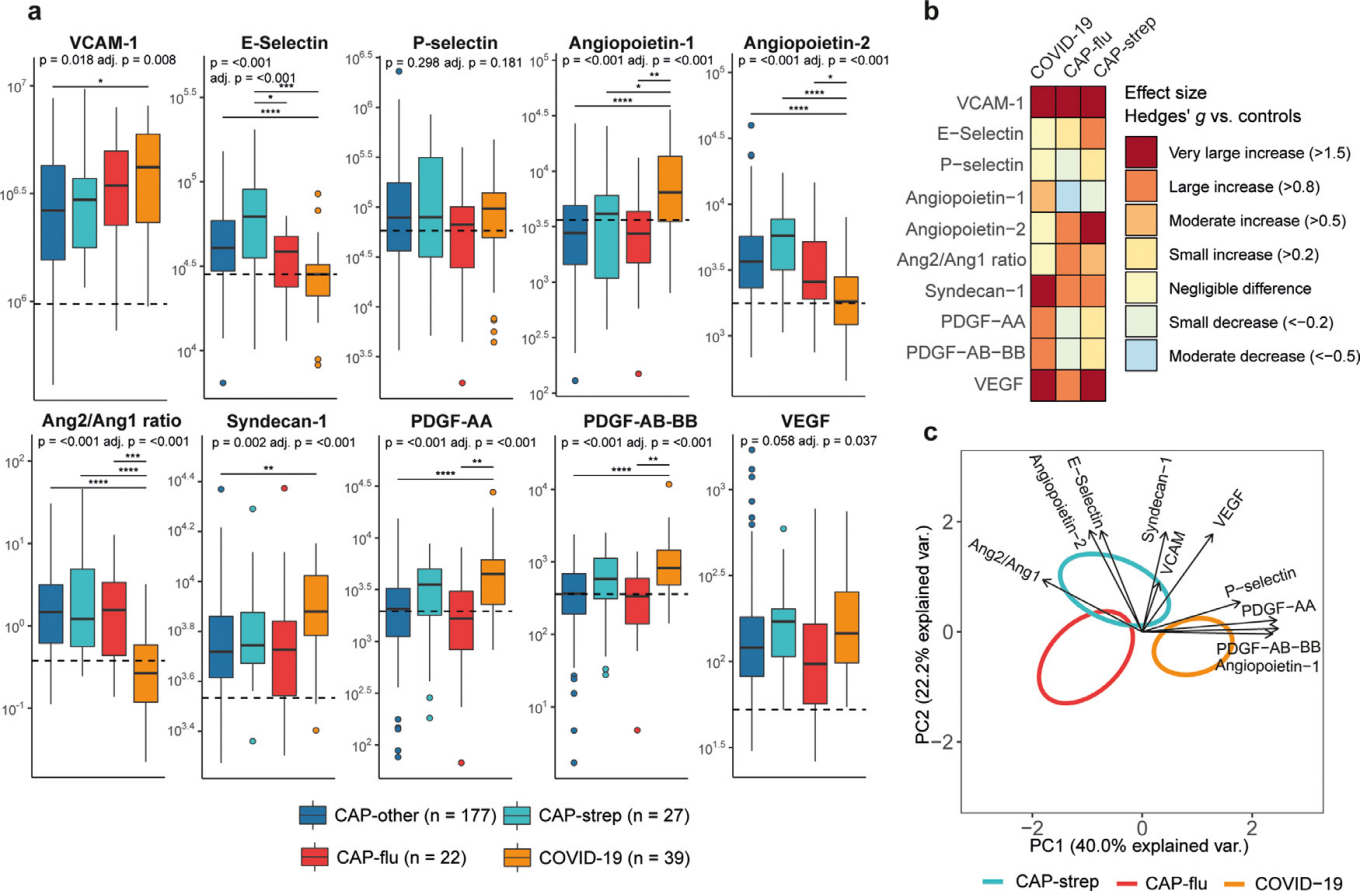
Biomarker levels reflective of vascular responses and function in patients with community-acquired pneumonia with different microbial aetiologies. **a)** Boxplots comparing markers of the endothelial response between the CAP groups. The Y-axis depicts biomarker concentrations in pg/ml (prior to Box-Cox transformation). The median value for control subjects is shown as a dashed line. The P-value listed in each boxplot is obtained from the White-adjusted ANOVA, the adjusted (adj.) P-value was obtained from the White-adjusted ANCOVA model including age, sex, and disease severity scores as covariates. The stars depict the significance of the post-hoc pairwise Games-Howell tests performed after a significant ANOVA. *P<0·05, **P<0·01, ***P<0·001, ****P<0·0001. Pairwise Tukey's post-hoc tests using the adjusted means from the ANCOVA for VEGF did not result significantly different groups. **b)** Heatmap depicting the Hedges’ *g* between the disease groups and controls for all biomarkers of the endothelial response. **c**) Principal component analysis of all biomarkers of the endothelial response. X-axis label shows the percentage of explained variance on principal component 1, Y-axis label shows the percentage of explained variance on principal component 2. The ellipse indicates the central 10% of the groups. The arrows indicate the direction (arrow orientation) and strength (arrow length) of the correlation between each marker and the principal components.

The occurrence of endotheliopathy has received much attention in severe COVID-19,^7^ whilst its presence in other CAPs has been investigated less extensively. We analysed soluble biomarkers indicative of endothelial cell activation, damage, and (dys)function. Seven markers were differentially abundant between CAP groups, wherein differences almost exclusively were driven by COVID-19; E-selectin was the only differentially abundant endothelial marker in non-COVID-19 CAP patients (higher CAP-strep than in CAP-flu, P<0·05) (Figure 3a+b). Remarkably, deviations between groups did not unequivocally point to increased endotheliopathy in COVID-19. Of the widely used endothelial cell activation markers VCAM-1 (vascular cell adhesion molecule) and E-selectin, VCAM-1 was higher, whilst E-selectin was lower in COVID-19 patients as compared with the non-COVID-19 CAP groups. Strikingly, the angiopoietin-2/1 ratio was elevated in all CAP groups except for COVID-19 (P<0·001 between COVID-19 and all other groups), suggesting that endothelial barrier integrity is more impaired in non-COVID-19 CAP than in COVID-19.^16^ Plasma syndecan-1 levels were higher in patients with COVID-19 than in other CAP groups, indicative of enhanced endothelial glycocalyx degradation.^17^ The platelet-derived growth factors (PDGF-AA and PDGF-AB-BB) – which function as pro-angiogenic and vascular remodelling factors^18^-were also specifically increased in patients with COVID-19.

The endothelial biomarkers clearly separated the pathogen-specific disease groups on the PCA plot (Figure 3c), with the angiopoietins, PDGFs and P-selectin driving the separation on PC1 of patients with COVID-19 from the others (ANOVA P<0·001, Games-Howell P<0·05 for COVID-19 versus CAP-strep and P<0·001 for COVID-19 versus CAP-flu), whereas PC2 separated viral from non-viral pneumonia mainly by syndecan-1 and E-selectin (ANOVA P<0·01, Games-Howell P<0·05 for CAP-flu versus CAP-strep, and P<0·01 for COVID-19 versus CAP-strep). Taken together, endothelial barrier integrity appeared relatively unperturbed in COVID-19 compared with other aetiologies of CAP, although markers of glycocalyx damage and angiogenesis were increased.

### Coagulation

**Figure 4:**
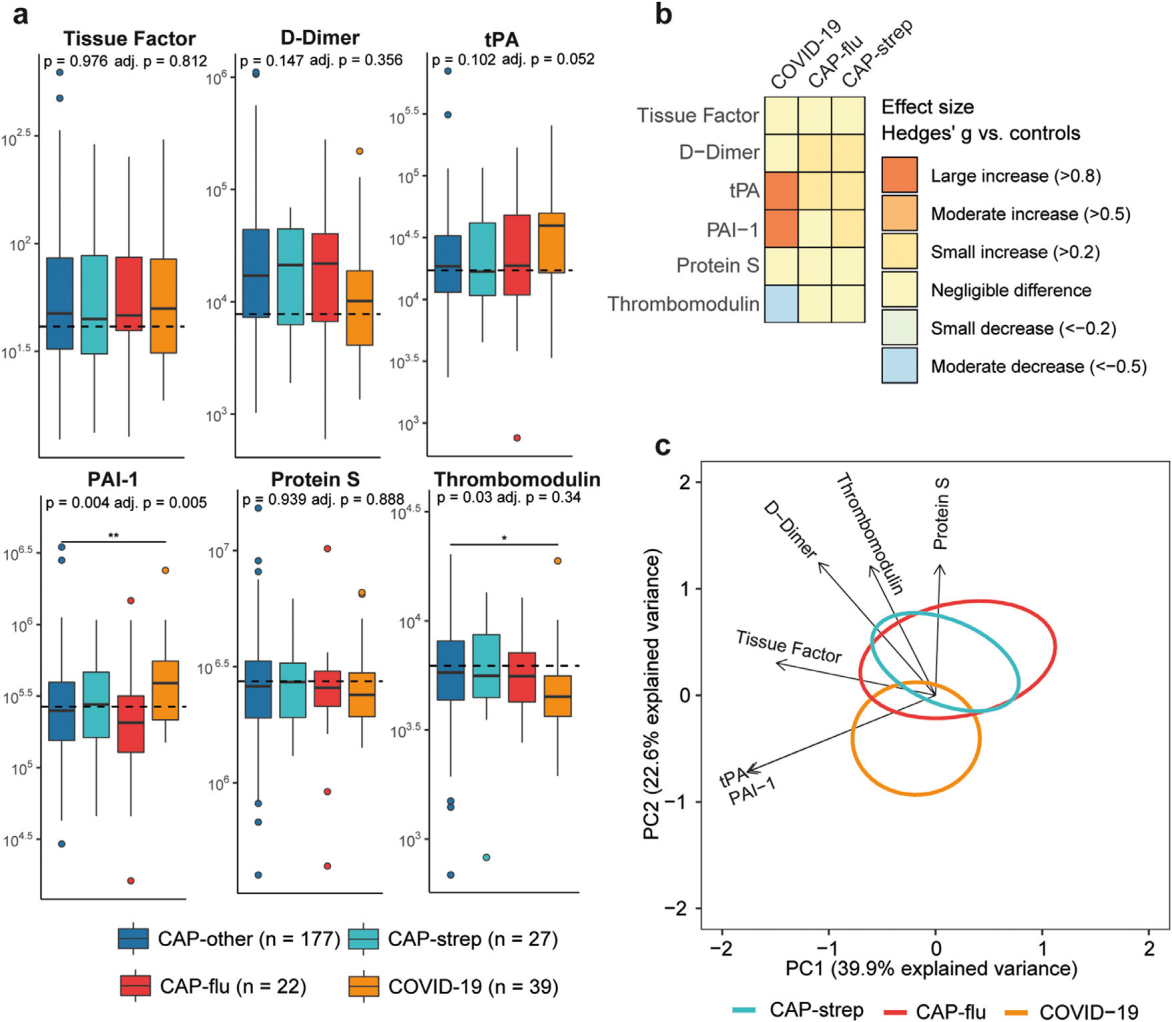
Biomarker levels reflective of coagulation in patients with community-acquired pneumonia with different microbial aetiologies. **a)** Boxplots comparing markers of coagulation between the CAP groups. The Y-axis depicts biomarker concentrations in pg/ml (prior to Box-Cox transformation). The median value for control subjects is shown as a dashed line. The P-value listed in each boxplot is obtained from the White-adjusted ANOVA, the adjusted (adj.) P-value was obtained from the White-adjusted ANCOVA model including age, sex, and disease severity scores as covariates. The stars depict the significance of the post-hoc pairwise Games-Howell tests performed after a significant ANOVA. *P<0·05, **P<0·01. **b)** Heatmap depicting the Hedges’ *g* between the disease groups and controls for all markers of coagulation. **c**) Principal component analysis of all markers of coagulation. X-axis label shows the percentage of explained variance on principal component 1, Y-axis label shows the percentage of explained variance on principal component 2. The ellipse indicates the central 10% of the groups. The arrows indicate the direction (arrow orientation) and strength (arrow length) of the correlation between each marker and the principal components.

Endothelial cell (dys)function is intricately linked to coagulation.^19^ However, soluble coagulation biomarkers showed only minor differences between disease groups (Figure 4a+b). Most notably, the plasma levels of tissue factor and D-dimer were not different between groups. When comparing patients with COVID-19 with other CAP groups, the plasma concentrations of PAI-1 (plasminogen activator inhibitor-1, an inhibitor of fibrinolysis) were higher (P<0·01 between COVID-19 and CAP-other), while the levels of thrombomodulin (an anticoagulant produced by endothelial cells) were lower, although the latter lost significance after adjusting for covariates (ANCOVA P=0·34). PCA confirmed that the disease groups could not clearly be separated based on multivariate analysis of coagulation markers (Figure 4c, PC1 not significant between groups, PC2 only significant between COVID-19 and CAP-strep with P<0·05). Altogether, these findings do not indicate large differences in coagulation activation between aetiologies of CAP.

### Systemic inflammation

**Figure 5:**
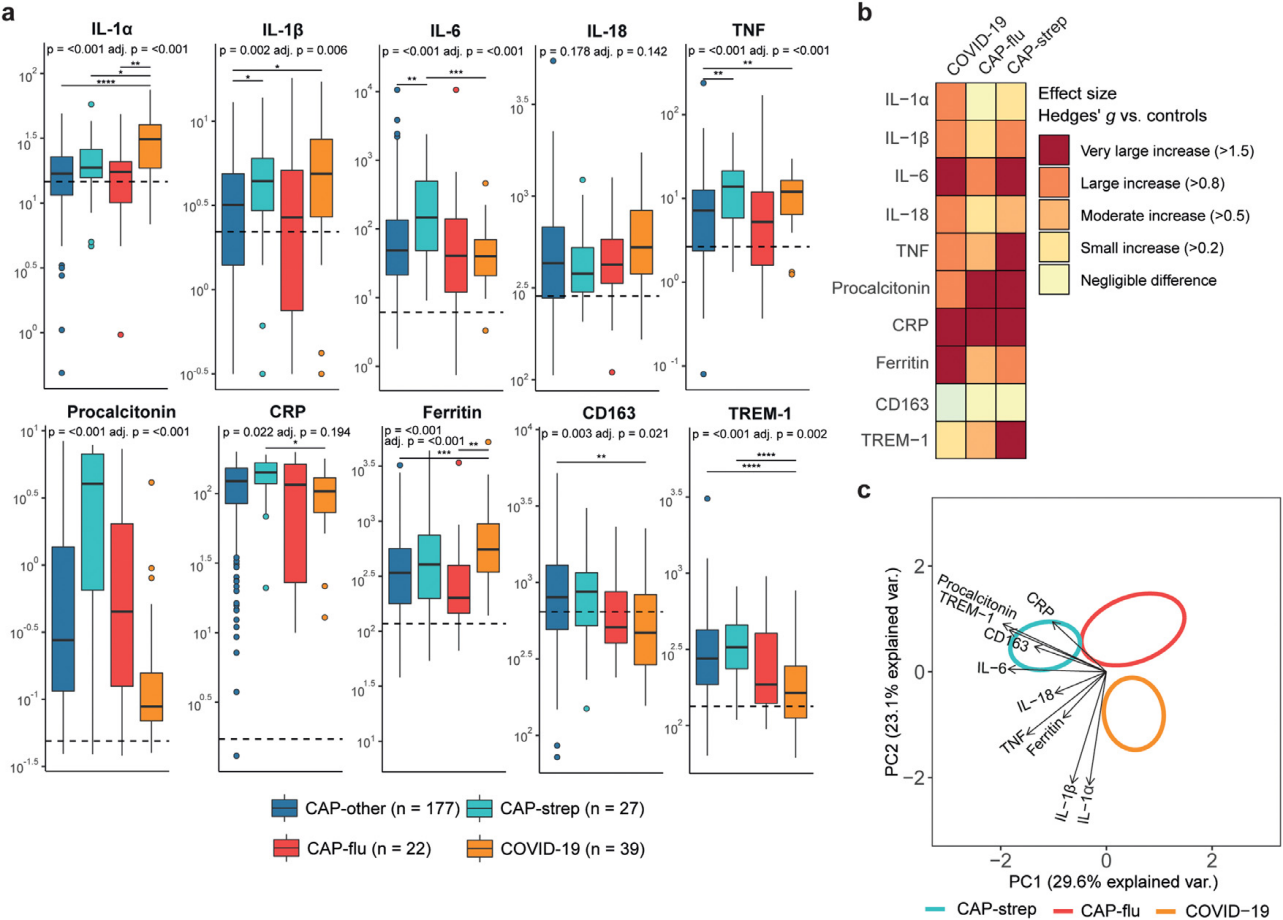
Biomarker levels reflective of systemic inflammation in patients with community-acquired pneumonia with different microbial aetiologies. **a)** Boxplots comparing markers of systemic inflammation between the CAP groups. The Y-axis depicts biomarker concentrations (prior to Box-Cox transformation) in pg/ml for all markers except for ferritin, procalcitonin, CD163 (ng/ml) and CRP (mg/L). The median value for control subjects is shown as a dashed line. The P-value listed in each boxplot is obtained from the White-adjusted ANOVA, the adjusted (adj.) P-value was obtained from the White-adjusted ANCOVA model including age, sex, and disease severity scores as covariates. The stars depict the significance of the post-hoc pairwise Games-Howell tests performed after a significant ANOVA. *P<0·05, **P<0·01, ***P<0·001, ****P<0·0001. **b)** Heatmap depicting the Hedges’ *g* between the disease groups and controls for all markers of systemic inflammation. **c**) Principal component analysis of all markers of systemic inflammation. X-axis label shows the percentage of explained variance on principal component 1, Y-axis label shows the percentage of explained variance on principal component 2. The ellipse indicates the central 10% of the groups. The arrows indicate the direction (arrow orientation) and strength (arrow length) of the correlation between each marker and the principal components.

Analyses of biomarkers reflecting systemic inflammation revealed stark differences between the disease groups (Figure 5a+b). IL-1α, IL-1β and ferritin were relatively increased in patients with COVID-19, particularly when compared with CAP-other and CAP-flu, whilst procalcitonin, IL-6 and CRP were highest in CAP-strep. CD163 and TREM-1 levels were relatively low in both viral CAP groups (CAP-flu and COVID-19), although only significant in the latter group. In the PCA analysis, principal component 1 (PC1) mainly separated viral (CAP-flu and COVID-19) from bacterial pneumonia (CAP-strep) and was driven by IL-6, CD163, TREM-1 and procalcitonin (Figure 5c, ANOVA P<0·0001, Games-Howell P<0·0001 for COVID-19 versus CAP-strep, P<0·01 for CAP-flu versus CAP-strep, COVID-19 versus CAP-flu not significant). PC2 separated COVID-19 patients from other forms of CAP due to elevated levels of IL-1α and IL-1β (PC2 scores for COVID-19 versus both other groups P<0·001, not significant between CAP-flu and CAP-strep).

### Immune checkpoint markers

**Figure 6:**
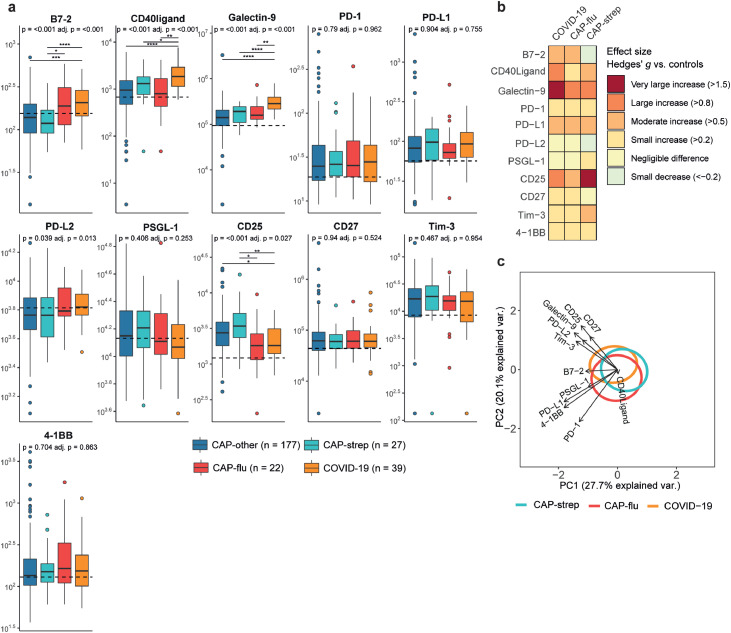
Immune checkpoint markers in patients with community-acquired pneumonia with different microbial aetiologies. **a)** Boxplots comparing immune checkpoint markers between the CAP groups. The Y-axis depicts biomarker concentrations in pg/ml (prior to Box-Cox transformation). The median value for control subjects is shown as a dashed line. The P-value listed in each boxplot is obtained from the White-adjusted ANOVA, the adjusted (adj.) P-value was obtained from the White-adjusted ANCOVA model including age, sex, and disease severity scores as covariates. The stars depict the significance of the post-hoc pairwise Games-Howell tests performed after a significant ANOVA. *P<0·05, **P<0·01, ***P<0·001, ****P<0·0001. **b)** Heatmap depicting the Hedges’ *g* between the disease groups and controls for all checkpoint markers. **c**) Principal component analysis of all immune checkpoint markers. X-axis label shows the percentage of explained variance on principal component 1, Y-axis label shows the percentage of explained variance on principal component 2. The ellipse indicates the central 10% of the groups. The arrows indicate the direction (arrow orientation) and strength (arrow length) of the correlation between each marker and the principal components.

Immune checkpoints are receptors expressed on immune cells that initiate suppressive or activating co-signalling pathways after binding their respective ligands.^20^ Plasma B7-2, the ligand for both CD28 and CTLA-4 (cytotoxic T lymphocyte antigen 4), was elevated in both CAP-flu and COVID-19 (Figure 6a+b). Interestingly, levels of B7-2 in CAP-other and CAP-strep appeared on par with, or even lower than non-infectious controls. CD40 ligand and galectin-9 were both distinctly increased in patients with COVID-19 (i.e. P<0·0001 for COVID-19 versus CAP-other). Galectin-9 is a ligand for TIM-3 (T cell immunoglobulin and mucin domain-containing protein 3), a cell surface receptor expressed on a range of cell types including several T cell subsets.^21^ CD25 (IL-2 receptor alpha chain), indicative of T cell activation,^22^ was lower in CAP-flu and COVID-19 than the other disease groups, but still higher than in non-infectious controls. Of note, the other checkpoint markers-such as PD-1 and PD-1 ligand-showed small to moderate increases, with little variation between the pathogen-specific groups. This was also reflected in the PCA plot, as the three disease groups mostly overlapped regarding checkpoint markers (Figure 6c, no significant differences).

### Subgroup and sensitivity-analyses

Finally, we explored the host response of other relevant subgroups within the cohort (Figure S1). A comparison of all patients with a confirmed viral infection-including COVID-19-versus all patients with a bacterial pathogen yielded results fairly similar to the COVID-19 versus all other CAP analysis (Figure S1a, Figure 1a). Removal of patients with COVID-19 from this viral group resulted in fewer significantly different biomarkers: only CXCL10 was higher in the viral group, while both PDGFs and P-selectin were most increased in the bacterial group (Figure S1b). When we compared non-COVID-19-viral patients to patients with COVID-19 (Figure S1c), we again observed a strong COVID-19 signature, mostly in line with the CAP-flu versus COVID-19 comparison in Figure 1d. We finally subdivided the bacterial CAP group into a gram-positive and gram-negative group, but this direct comparison yielded only one significantly different biomarker (CXCL10 higher in gram-negative CAP, Figure S1d), possibly due to low sample size and within-group heterogeneity.

Next, to assess the robustness of the results in this investigation we performed several sensitivity-analyses. In the first, we removed five patients in whom the pathogen diagnosis was ambivalent (three co-infected patients and two patients with PCR-negative COVID-19), which had a negligible impact on the results (see Supplemental Table 6 for the untargeted analyses equivalent to Figure 1, and Supplemental Table 7 for the between-group differences and post-hoc tests per biomarker per pathophysiological domain as in Figure 2-6). In the second sensitivity analysis we left out all patients with an immune deficiency (44 patients). Again, this resulted in negligible differences in the untargeted analyses, and a small number of differences in the analyses per domain, such as IL-7, CD25 and thrombomodulin. For the next sensitivity-analysis, we removed all patients that were given high-dose systemic glucocorticoids prior to, or upon hospital admission (42 patients). This resulted in a loss of significance for a number of comparisons involving CAP-flu (possibly reflecting a loss of statistical power due to lower sample size, as 15/22 patients remain in the CAP-flu group). Finally, we assessed the effect of BMI, comorbidities and inclusion centre (see Supplemental Table 8). Addition of BMI and the total number of comorbidities to the ANCOVA model resulted in the loss of significance of one marker (CD25). After a multilevel pooled analysis, in which we added the inclusion site as a level in the model, tPA and IL-7-which were borderline significant before-became significant. Combination of the multilevel analysis with the addition of BMI and comorbidities as covariates resulted in the loss of significance for CD25, while IL-7 became significant. Collectively, these sensitivity analyses indicate that the host response differences between patients with different aetiologies of CAP were robust.

## Discussion

We utilised an extensive panel of soluble plasma biomarkers to analyse the host response of patients hospitalized for CAP with different microbial aetiologies, and revealed common and distinct features across several pathophysiological domains. Biomarker profiles reflecting activation and function of the vasculature suggested divergent characteristics in COVID-19 as compared with non-COVID-19 CAPs. COVID-19 was associated with seemingly unperturbed barrier integrity (angiopoietin-2/1 ratio), enhanced glycocalyx degradation (syndecan-1) and elevated levels of angiogenic factors (PDGFs). Notably, the extent of coagulation activation, implicated in the relatively high incidence of thromboembolic complications in COVID-19,^23^ was not different between CAP groups (tissue factor, D-dimer), in spite of evidence of increased systemic inflammation in COVID-19 (ferritin). Some biomarkers confirmed previous reports on distinctive responses in CAP caused by different microorganisms, including strongly increased procalcitonin levels in CAP-strep and elevated concentrations of mediators implicated in antiviral responses in COVID-19,^12^^,^^15^ providing assurance that our cohort is able to expose pathophysiological distinctions across different CAP aetiologies. These results yield insight into the pathophysiology of CAP in general and may guide identification of targetable host response aberrations in CAPs caused by specific pathogens.

Angiopoietin-1 and angiopoietin-2 have diverging effects on endothelial integrity and vascular permeability, due to their opposing effects on the tyrosine kinase receptor Tie2.^16^ A low angiopoietin-2/1 ratio is thought to reflect a protective endothelial phenotype: angiopoeitin-1 has been shown to prevent vascular permeability, whereas angiopoietin-2 typically promotes inflammation, apoptosis and vascular leakage.^16^^,^^24^ A high angiopoietin-2/1 ratio strongly correlates with mortality in critically ill patients with acute lung injury,^25^ and high angiopoietin-2 has been associated with a worse outcome in COVID-19.^26^ In line with our findings, angiopoietin-2/1 ratios were lower in critically ill COVID-19 patients compared with non-COVID-19 patients in the intensive care unit.^27^ The less disturbed endothelial profile of patients with COVID-19, relative to other CAPs, was further illustrated by normal levels of E-selectin and the increase in PDGFs. PDGFs can be produced by many cell types, including endothelial cells, and stimulate angiogenesis and cell proliferation.^18^ *In vitro*, PDGF stimulation of vascular smooth muscle cells was shown to decrease angiopoietin-2 levels, possibly indicating a protective mechanism.^28^ Increased levels of PDGFs and angiopoietin-1 were associated with a favourable outcome in children with severe bacterial infection.^29^ Taken together, these findings may suggest that endothelial inflammation and disturbed barrier function play a larger role in non-COVID-19 CAP than in COVID-19.

COVID-19 was associated with higher plasma syndecan-1 levels as compared with other CAPs. Syndecan-1 is a glycocalyx component with protective and inflammation attenuating effects in cell-bound form.^30^ Increased shedding of syndecan-1 has been linked to higher disease severity in patients with sepsis.^17^ An intact glycocalyx is important for maintaining the constitutive anticoagulant properties of the endothelial cell surface.^16^ Likewise, the higher circulating levels of thrombomodulin in COVID-19-although non-significant after removal of immune deficient patients-may reflect augmented shedding from the endothelial cell surface, which is expected to result in a loss of its cell-associated anticoagulant properties.^31^ Nonetheless, plasma coagulation markers tissue factor and D-dimer were similar in different CAP groups. PAI-1, a major inhibitor of the fibrinolytic system, was significantly increased in COVID-19 compared with other CAP groups. In agreement, a recent study reported increased PAI-1 activity in patients with COVID-19 when compared with patients with sepsis.^32^ In our study D-dimer was relatively low in patients with COVID-19 (albeit not significantly different). This is in line with previous comparisons between patients with COVID-19 and sepsis,^32^^,^^33^ although other studies reported increased D-dimer in COVID-19 when compared with other CAPs.^34^^,^^35^ Together, our data may indicate that high PAI-1 levels could limit fibrin degradation and subsequent D-dimer release, resulting in relatively low D-dimer levels in COVID-19.

Immune checkpoint markers showed several differences between CAP groups. Galectin-9 was strikingly increased in patients with COVID-19. Galectin-9 is expressed by many cell types, such as neutrophils, T cells and various antigen-presenting cells, and is ascribed a wide range of immunomodulatory functions.^36^ It induces apoptosis of Th1 cells through binding with the TIM-3 receptor, and is overall regarded as an immunosuppressive molecule for adaptive immune cells.^20^ Specifically, binding of galectin-9 to TIM-3 is thought to have strong suppressive effects on effector T cells, and inhibition of this axis boosted antiviral responses in an influenza mouse model.^37^ The observation of high B7-2 and low CD25 levels-although CD25 differences became non-significant after adjustment for BMI, comorbidities and site-in both CAP-flu and COVID-19 when compared with CAP-other and CAP-strep might indicate a common viral effect on T cell activation. B7-2 is the ligand for CTLA4 and CD28, with either suppressive or activating effects respectively.^20^ CD25 expression is used to characterize regulatory T cells and is indicative of T cell activation.^22^

Importantly, COVID-19 patients were enrolled before dexamethasone became standard of care, which allowed us to compare the host response between COVID-19 and other forms of CAP without the likely strong confounding effects of this drug. To account for the influence of other variables (with sufficient data in each subgroup) in the relationship between pathogen groups and host response biomarkers, we adjusted comparisons of individual biomarkers for age, sex, and disease severity, which resulted in negligible differences in between-group p-values. Sensitivity analyses pertaining to coinfections, high-dose corticosteroid usage, immunocompromised status, BMI, comorbidities and inclusion centre further underlined the robustness of the results. As the vast majority of patients were included within 24 hours, we did not further investigate the effect of time between sampling and admission, although this may explain a small part of variance in the data. Furthermore, this study did not interfere with clinical diagnostics, so it is possible that unidentified bacterial infections, or viral/bacterial co-infections exist in this cohort, which may influence the pathogen-specific comparisons. Still, unidentified pathogens are more likely to dilute rather than exaggerate differences between CAP subgroups, and it does not limit the generalisability of our results as these groups reflect the current clinical reality of microbiological testing. Statistical power for between-subgroup comparisons may also be limited by the modest sample size in this study.

In response to the unique challenges posed by the COVID-19 pandemic, many high-quality trials have been performed in a very short timeframe. The number of immunomodulatory trials-enabled by unprecedented incentive and resources-in COVID-19 has rapidly surpassed all efforts in other aetiologies of CAP. The data in our study may inform future therapeutic strategies that aim to repurpose COVID-19 treatments for other CAP (sub)groups. For example, several studies explored the benefit of reducing vascular permeability and capillary leakage in COVID-19, and treatment with the tyrosine-kinase inhibitor imatinib resulted in improved outcome.^38^ As our current data suggest that the endothelial barrier integrity is most disturbed in non-COVID-19 CAP, it may be interesting to expand this rationale to CAP aetiologies other than COVID-19. Another example of how biomarkers can instruct treatment decisions is the recent finding that hyperferritinemia (in our cohort most prominent in COVID-19) is a predictor of a favourable response to treatment with recombinant IL-1 receptor antagonist in patients with COVID-19.^39^ Notwithstanding, translating COVID-19 specific therapies to other infections is challenging, exemplified by the harm that is done by treating influenza pneumonia with dexamethasone.^40^

In summary, our investigation offers a nuanced assessment of the systemic host response in different aetiologies of CAP, and gives insight into shared and distinct pathophysiological mechanisms that may guide therapy in COVID-19 as well as in the wider CAP population.

## Contributors

ARS, TDYR: Conceptualisation, investigation, data collection, data curation, formal analysis, methodology, project administration, visualisation, writing-original draft, and writing-review & editing. TSRE: investigation, data curation. VL, JdB, CvL, MS, LP, BWH, XB, MK, IB, RD, DRF: investigation, data collection, project administration, writing-review & editing. PWBN, JS, JMP, BPS, WJW, TvP: funding acquisition, supervision, writing-review & editing. ARS, TDYR, TSRE, TVDP and WJW have directly accessed and verified the underlying data reported in the manuscript. All authors have read and approved the final version of the manuscript.

## Declaration of interests

JS was on the Data Safety Monitoring Board of the Pointer trial (ISRCTN33682933), and is the Vice President of the European Society of Gastrointestinal and Abdominal Radiology. All other authors declare no conflict of interests.

## Appendix Supplementary materials

Image, application 1

Image, application 2

Image, application 3

Image, application 4

Image, application 5

Image, application 6
