# Supplementary material for: The host response in different aetiologies of community-acquired pneumonia
Source: eBioMedicine. 2022 Jun 1;81:104082. doi: 10.1016/j.ebiom.2022.104082 (PMC9155985; doi:10.1016/j.ebiom.2022.104082)
Supplement: Supplementary file 1 [file mmc1.docx]

**Figure S1. Subgroup analysis of the host response during CAP.**

**a)** Volcano plot comparing plasma biomarkers between patients with viral CAP (SARS-CoV-2, influenza A and B viruses, respiratory syncytial virus, parainfluenza virus, human metapneumovirus, coronavirus, rhinovirus, n=79) and patients with bacterial CAP (*Streptococcus pneumoniae*, *Haemophilus influenzae, Staphylococcus aureus*, *Klebsiella pneumoniae, Pseudomonas aeruginosa, Mycoplasma pneumoniae, Legionella, Rothia dentocariosa, Escherichia coli,* n=60). X-axis depicts the fold change of Box-Cox transformed values between groups, Y-axis depicts the Benjamini-Hochberg adjusted P-value. **b)** Volcano plot comparing non-COVID-19-viral CAP (n=42) with bacterial CAP (n=60). **c)** Volcano plot comparing non-COVID-19-viral CAP (n=42) with COVID-19 (n=39). **d)** Volcano plot comparing gram-negative bacterial CAP (*Haemophilus influenzae*, *Pseudomonas aeruginosa*, *Klebsiella pneumoniae*, *Legionella, Escherichia coli*, n=27) with gram-positive bacterial CAP (*Streptococcus pneumoniae*, *Staphylococcus aureus, Rothia dentocariosa*, n=32). CAP, community-acquired pneumonia.
