# Supplementary material for: The host response in different aetiologies of community-acquired pneumonia
Source: eBioMedicine. 2022 Jun 1;81:104082. doi: 10.1016/j.ebiom.2022.104082 (PMC9155985; doi:10.1016/j.ebiom.2022.104082)
Supplement: Supplementary file 2 [file mmc2.docx]

| **Supplemental Table 1· Overview of missing data in clinical variables·** | | | | |
| --- | --- | --- | --- | --- |
|  | **CAP-other** | **CAP-strep** | **CAP-flu** | **COVID-19** |
|  | **(n = 177)** | **(n = 27)** | **(n = 22)** | **(n = 39)** |
| **DEMOGRAPHICS** |  |  |  |  |
| Age (years) | 0 | 0 | 0 | 0 |
| Gender (male) | 0 | 0 | 0 | 0 |
| BMI | 15 (8·5) | 0 | 2 (9·1) | 1 (2·6) |
| **CHRONIC COMORBIDITIES** |  |  |  |  |
| Immune deficiency | 0 | 0 | 0 | 0 |
| COPD | 0 | 0 | 0 | 0 |
| Asthma | 0 | 0 | 0 | 0 |
| Congestive heart failure | 0 | 0 | 0 | 0 |
| Myocardial infarction | 0 | 0 | 0 | 0 |
| Stroke | 0 | 0 | 0 | 0 |
| Diabetes mellitus (type 1 or 2) | 0 | 0 | 0 | 0 |
| Chronic kidney disease | 0 | 0 | 0 | 0 |
| **LABORATORY TESTS** |  |  |  |  |
| Leukocytes x10^9^/L | 1 (0·6) | 0 | 0 | 0 |
| Neutrophils x10^9^/L | 38 (21·5) | 6 (22·2) | 4 (18·2) | 1 (2·6) |
| Lymphocytes x10^9^/L | 33 (18·6) | 5 (18·5) | 4 (18·2) | 1 (2·6) |
| Platelets x10^9^/L | 5 (2·8) | 1 (3·7) | 0 | 0 |
| Creatinine, µmol/L | 12 (6·8) | 0 | 0 | 0 |
| **VITAL SIGNS AND DISEASE SEVERITY** |  |  |  |  |
| Temperature, degrees °C | 0 | 0 | 0 | 0 |
| Respiratory rate | 5 (2·8) | 0 | 1 (4·5) | 0 |
| Heart rate | 0 | 0 | 0 | 0 |
| Mean arterial pressure | 0 | 0 | 0 | 0 |
| Modified Early Warning Score | 5 (2·8) | 0 | 1 (4·5) | 0 |
| Pneumonia Severity Index | 3 (1·7) | 2 (7·4) | 0 | 0 |
| CURB-65 | 11 (6·2) | 2 (7·4) | 2 (9·1) | 0 |
| qSOFA | 1 (0·6) | 0 | 0 | 0 |
| **DISEASE COURSE** |  |  |  |  |
| Duration of symptoms prior to admission (days)* | 50 (28·2) | 3 (11·1) | 7 (31·8) | 0 |
| Increase of corticosteroids upon admission | 0 | 0 | 0 | 0 |
| ICU admission (at any point during admission) | 0 | 0 | 0 | 0 |
| Time to clinical stability or discharge | 6 (3·4) | 0 | 0 | 0 |
| Hospital length of stay (days) | 0 | 0 | 0 | 0 |
| 28-day mortality | 0 | 0 | 0 | 0 |
| CAP=community-acquired pneumonia; COPD=chronic obstructive pulmonary disease; bpm=breaths/beats per minute; MAP=mean arterial pressure; CURB-65=confusion, blood urea nitrogen, respiratory rate, blood pressure, age 65 or older; MEWS=modified early warning score; PSI=pneumonia severity index; qSOFA=quick sequential organ failure assessment score; LOS=length of stay. | | | | |
| Data are presented as count (percentage) and compared using Fisher’s exact test. | | | | |
| *Date of start of symptoms was not available in the OPTIMACT study | | | | |

| **Supplemental Table 2. Assay overview.** | | |  |  |  |
| --- | --- | --- | --- | --- | --- |
| **Analyte** | **Assay** | **% below LLOQ** | **% above ULOQ** | **% within detection range** | **Reason removed** |
| **Cell-mediated antiviral response** | | |  |  |  |
| β2-Microglobulin | Luminex | 0 | 0 | 100 |  |
| CD40 ligand | Luminex | 4 | 0 | 96 |  |
| Granzyme B | Luminex | 9 | 0 | 91 |  |
| IFNα | Luminex | 2 | 0 | 98 |  |
| IFNγ | Luminex | 10 | 0 | 90 |  |
| IL-2 | Luminex | 8 | 0 | 92 |  |
| IL-7 | Luminex | 0 | 0 | 100 |  |
| CXCL10 | Luminex | 0 | 0 | 100 |  |
| **Vascular responses and function** | | |  |  |  |
| VCAM-1 | Luminex | 0 | 0 | 100 |  |
| E-Selectin | Luminex | 0 | 0 | 100 |  |
| P-selectin | CBA | 0 | 0 | 100 |  |
| Angiopoietin-1 | Luminex | 0 | 0 | 100 |  |
| Angiopoietin-2 | Luminex | 0 | 0 | 100 |  |
| Syndecan-1 | Luminex | 0 | 0 | 100 |  |
| PDGF-AA | Luminex | 0 | 2 | 98 |  |
| PDGF-AB-BB | Luminex | 0 | 0 | 100 |  |
| VEGF | Luminex | 0 | 0 | 100 |  |
| **Coagulation** |  |  |  |  |  |
| Tissue Factor | CBA | 0 | 0 | 100 |  |
| D-Dimer | CBA | 0 | 0 | 100 |  |
| tPA | CBA | 0 | 0 | 100 |  |
| PAI-1 | CBA | 0 | 0 | 100 |  |
| Protein S | Luminex | 0 | 0 | 100 |  |
| Thrombomodulin | Luminex | 0 | 0 | 100 |  |
| **Systemic inflammation** |  |  |  |  |  |
| IL-1α | Luminex | 0 | 0 | 100 |  |
| IL-1β | Luminex | 9 | 0 | 91 |  |
| IL-6 | Luminex | 0 | 0 | 100 |  |
| IL-18 | Luminex | 0 | 0 | 100 |  |
| Procalcitonin | Luminex | 0 | 0 | 100 |  |
| CRP | Luminex | 1 | 0 | 99 |  |
| Ferritin | Luminex | 0 | 0 | 100 |  |
| TNF | Luminex | 10 | 0 | 90 |  |
| TREM-1 | Luminex | 0 | 0 | 100 |  |
| CD163 | Luminex | 0 | 0 | 100 |  |
| **Immune checkpoint markers** | |  |  |  |  |
| B7-2 | CBA | 0 | 0 | 100 |  |
| CD40 ligand | Luminex | 4 | 0 | 96 |  |
| Galectin-9 | CBA | 0 | 0 | 100 |  |
| PD-1 | CBA | 5 | 0 | 95 |  |
| PD-L1 | CBA | 1 | 0 | 99 |  |
| PD-L2 | CBA | 0 | 0 | 100 |  |
| PSGL-1 | CBA | 0 | 0 | 100 |  |
| CD25 | CBA | 0 | 0 | 100 |  |
| CD27 | CBA | 0 | 0 | 100 |  |
| Tim-3 | CBA | 2 | 0 | 98 |  |
| 4-1BB | CBA | 0 | 0 | 100 |  |
| **Other cytokines** |  |  |  |  |  |
| IL-1RA | Luminex | 0 | 0 | 100 |  |
| IL-3 | Luminex | 18 | 0 | 82 |  |
| IL-4 | Luminex | 6 | 0 | 94 |  |
| IL-5 | Luminex | 16 | 0 | 84 |  |
| IL-8 | Luminex | 0 | 0 | 100 |  |
| IL-10 | Luminex | 0 | 0 | 100 |  |
| IL-13 | Luminex | 2 | 0 | 98 |  |
| IL-15 | Luminex | 5 | 0 | 95 |  |
| IL-17A | Luminex | 5 | 0 | 95 |  |
| IL-33 | Luminex | 3 | 0 | 97 |  |
| **Other chemokines** |  |  |  |  |  |
| EGF | Luminex | 1 | 0 | 99 |  |
| CCL11 | Luminex | 0 | 0 | 100 |  |
| Fractalkine | Luminex | 0 | 0 | 100 |  |
| G-CSF | Luminex | 0 | 0 | 100 |  |
| GM-CSF | Luminex | 0 | 0 | 100 |  |
| CXCL1 | Luminex | 0 | 0 | 100 |  |
| CXCL2 | Luminex | 0 | 0 | 100 |  |
| IL-8 | Luminex | 0 | 0 | 100 |  |
| CCL2 | Luminex | 0 | 0 | 100 |  |
| CCL3 | Luminex | 3 | 0 | 97 |  |
| CCL4 | Luminex | 2 | 0 | 98 |  |
| CCL19 | Luminex | 0 | 0 | 100 |  |
| CCL20 | Luminex | 0 | 0 | 100 |  |
| CCL5 | Luminex | 0 | 0 | 100 |  |
| TGF-α | Luminex | 0 | 0 | 100 |  |
| CCL22 | Luminex | 0 | 0 | 100 |  |
| **Other** |  |  |  |  |  |
| SP-D | Luminex | 0 | 0 | 100 |  |
| TRAIL | Luminex | 2 | 0 | 98 |  |
| Flt-3ligand | Luminex | 0 | 0 | 100 |  |
| Factor-IX | Luminex | 0 | 0 | 100 |  |
| **Removed** |  |  |  |  |  |
| IL-12p70 | Luminex | 83 | 0 | 17 | >40% lower than LLOQ |
| IL-17E | Luminex | 48 | 0 | 52 | >40% lower than LLOQ |
| FGF-basic | Luminex | 47 | 0 | 53 | >40% lower than LLOQ |
| IFNβ | Luminex | 60 | 0 | 40 | >40% lower than LLOQ |
| LAG-3 | CBA | 48 | 0 | 52 | >40% lower than LLOQ |
| TGF-β1 | CBA | 95 | 0 | 5 | >40% lower than LLOQ |
| CTLA-4 | CBA | 55 | 0 | 45 | >40% lower than LLOQ |

| **Supplemental Table 3. Clinical characteristics of all patients and control subjects.** | | | |
| --- | --- | --- | --- |
|  | **All patients** | **Controls** | **p-value** |
|  | **(n = 265)** | **(n = 28)** |  |
| **DEMOGRAPHICS** |  |  |  |
| Age , years | 66·2 (15·7) | 65·4 (12·6) | 0·80 |
| Sex, male | 148 (55·8) | 16 (57·1) | >0·99 |
| Body mass index | 26·4 (5·9) | 28·6 (7·8) | 0·07 |
| **CHRONIC COMORBIDITIES** |  |  |  |
| Immune deficiency | 44 (16·6) | 0 (0·0) | 0·01 |
| Chronic obstructive pulmonary disease | 71 (26·8) | 5 (17·9) | 0·37 |
| Asthma | 23 (8·7) | 0 (0·0) | 0·14 |
| Congestive heart failure | 20 (7·5) | 3 (10·7) | 0·47 |
| Myocardial infarction | 39 (14·7) | 2 (7·1) | 0·39 |
| Stroke | 24 (9·1) | 0 (0·0) | 0·15 |
| Diabetes mellitus (type 1 or 2) | 68 (25·7) | 6 (21·4) | 0·82 |
| Chronic kidney disease | 28 (10·6) | 2 (7·1) | 0·75 |
| Continuous data are presented as mean (standard deviation) or median [interquartile range], and compared using a two-sided *t*-test or two-sided Wilcoxon rank-sum test, respectively. Categorical data are presented as count (percentage) and compared using Fisher’s exact test. | | | |

| **Supplemental Table 4. Clinical characteristics of non-COVID-19 CAP and COVID-19 patients.** | | | |
| --- | --- | --- | --- |
|  | **Non-COVID-19 CAP** | **COVID-19** | **p-value** |
|  | **(n = 226)** | **(n = 39)** |  |
| **DEMOGRAPHICS** |  |  |  |
| Age (years) | 67·2 (16·1) | 60·5 (11·4) | 0·01 |
| Gender (male) | 128 (56·6) | 20 (51·3) | 0·60 |
| BMI | 25·7 (5·5) | 30·3 (6·9) | <0·01 |
| **CHRONIC COMORBIDITIES** |  |  |  |
| Immune deficiency | 42 (18·6) | 2 (5·1) | 0·04 |
| COPD | 69 (30·5) | 2 (5·1) | <0·01 |
| Asthma | 20 (8·8) | 3 (7·7) | >0·99 |
| Congestive heart failure | 19 (8·4) | 1 (2·6) | 0·33 |
| Myocardial infarction | 37 (16·4) | 2 (5·1) | 0·09 |
| Stroke | 21 (9·3) | 3 (7·7) | >0·99 |
| Diabetes mellitus (type 1 or 2) | 58 (25·7) | 10 (25·6) | >0·99 |
| Chronic kidney disease | 26 (11·5) | 2 (5·1) | 0·39 |
| **LABORATORY TESTS*** |  |  |  |
| Leukocytes x10^9^/L | 11·8 [8·6, 15·3] | 6·9 [5·7, 9·0] | <0·01 |
| Neutrophils x10^9^/L | 9·3 [6·1, 12·3] | 5·1 [3·9, 6·9] | <0·01 |
| Lymphocytes x10^9^/L | 0·94 [0·60, 1·47] | 0·97 [0·72, 1·40] | 0·87 |
| Platelets x10^9^/L | 246 (116) | 261 (108) | 0·47 |
| Creatinine, µmol/L | 89 [68, 119] | 88[71, 102] | 0·44 |
| **VITAL SIGNS AND DISEASE SEVERITY*** |  |  |  |
| Temperature, degrees °C | 38·2 (1·13) | 37·7 (1·20) | <0·01 |
| Respiratory rate | 22 [18, 27] | 23 [20, 27] | 0·06 |
| Heart rate | 98 [85, 110] | 94 [80·5, 107·5] | 0·32 |
| Mean arterial pressure | 96 (17) | 96 (15) | 0·78 |
| Modified Early Warning Score | 3 [2, 5] | 3 [2·5, 4] | 0·67 |
| Pneumonia Severity Index | 4 [3, 4] | 3 [2, 3] | <0·01 |
| CURB-65 | 2 [1, 2] | 1 [0, 2] | 0·01 |
| qSOFA | 1 [0, 1] | 1 [0, 1] | 0·22 |
| **DISEASE COURSE** |  |  |  |
| Duration of symptoms prior to admission (days) | 4 [2, 7] | 8 [5, 10] | <0·01 |
| Increase of corticosteroids upon admission | 40 (17·7) | 2 (5·1) | 0.06 |
| ICU admission (at any point during admission) | 20 (8·8) | 6 (15·4) | 0·24 |
| Time to clinical stability† or discharge | 3 [2, 6] | 4 [3, 7] | 0·09 |
| Hospital length of stay (days) | 4 [3, 9] | 4 [3, 7·5] | 0·97 |
| 28-day mortality | 12 (5·3) | 5 (12·8) | 0·09 |
| CAP=community-acquired pneumonia; COPD=chronic obstructive pulmonary disease; bpm=breaths/beats per minute; MAP=mean arterial pressure; CURB-65=confusion, blood urea nitrogen, respiratory rate, blood pressure, age 65 or older; MEWS=modified early warning score; PSI=pneumonia severity index; qSOFA=quick sequential organ failure assessment score; LOS=length of stay. | | | |
| Continuous data are presented as mean (standard deviation) or median [interquartile range], and compared using a two-sided ANOVA or two-sided Kruskal-Wallis test, respectively. Categorical data are presented as count (percentage) and compared using Fisher’s exact test. | | | |
| *****Measured upon presentation to the emergency department | |  |  |
| †Defined as the modified Halm's criteria: temperature ≤37·2^°^C, heart rate ≤100 bpm, systolic blood pressure ≤90 mmHg, respiratory rate ≤ 24 bpm, and oxyen saturation ≥90% for the entire day. | | | |

| **Supplemental Table 5. Specific host response markers.** | | | |  |  |  |  |  |
| --- | --- | --- | --- | --- | --- | --- | --- | --- |
| **COVID-19 specific response** |  |  |  |  | **Non-COVID-19 CAP specific response** | |  |  |
| **Marker** | **Adjusted P** | **Log2FC** | **Direction** |  | **Marker** | **Adjusted P** | **Log2FC** | **Direction** |
| Angiopoietin-1 | 0,026117647 | 0,559682 | Up |  | CCL22 | 0,002763692 | -0,55854 | Down |
| Thrombomodulin | 0,01098383 | -0,51231 | Down |  | E-Selectin | 0,000435 | 0,81559 | Up |
| CD40 ligand | 1,02E-05 | 1,284797 | Up |  | TREM-1 | 6,12E-07 | 1,050935 | Up |
| EGF | 0,014544 | 0,578847 | Up |  | Angiopoietin-2 | 5,75E-09 | 1,089117 | Up |
| Flt-3Ligand | 0,014544 | 0,514871 | Up |  | CCL11 | 0,0072 | -0,50878 | Down |
| Granzyme B | 1,38E-06 | 1,310555 | Up |  | TRAIL | 1,48E-07 | -1,11311 | Down |
| CXCL1 | 0,002829767 | 0,589144 | Up |  | D-Dimer | 0,030763636 | 0,448278 | Up |
| CXCL2 | 0,040133333 | 0,556285 | Up |  | Tim-3 | 0,0020592 | 0,558243 | Up |
| IFNα | 0,000682839 | 0,87691 | Up |  |  |  |  |  |
| IFNγ | 1,85E-05 | 1,307893 | Up |  |  |  |  |  |
| IL-1α | 0,000963 | 0,957079 | Up |  |  |  |  |  |
| IL-1β | 0,001072216 | 0,911826 | Up |  |  |  |  |  |
| IL-2 | 0,002451429 | 0,804358 | Up |  |  |  |  |  |
| IL-3 | 0,0001065 | 1,020647 | Up |  |  |  |  |  |
| IL-4 | 0,007136 | 0,713952 | Up |  |  |  |  |  |
| IL-5 | 0,035049057 | 0,550117 | Up |  |  |  |  |  |
| IL-13 | 0,001267579 | 0,813792 | Up |  |  |  |  |  |
| IL-17A | 0,001072216 | 0,843595 | Up |  |  |  |  |  |
| IL-33 | 1,80E-05 | 1,145976 | Up |  |  |  |  |  |
| CCL2 | 1,24E-06 | 0,939523 | Up |  |  |  |  |  |
| CCL3 | 0,0006672 | 0,85674 | Up |  |  |  |  |  |
| CCL4 | 0,000282857 | 0,730811 | Up |  |  |  |  |  |
| PDGF-AA | 0,000451862 | 0,88859 | Up |  |  |  |  |  |
| PDGF-AB-BB | 0,0015894 | 0,84835 | Up |  |  |  |  |  |
| CCL5 | 0,035049057 | 0,550575 | Up |  |  |  |  |  |
| TGF-α | 0,000113262 | 1,038124 | Up |  |  |  |  |  |
| tPA | 0,001072216 | 0,817406 | Up |  |  |  |  |  |
| PAI-1 | 0,000282667 | 0,777351 | Up |  |  |  |  |  |
| B7-2 | 0,007591304 | 0,597874 | Up |  |  |  |  |  |
