# Supplementary figures and images for: The host response in different aetiologies of community-acquired pneumonia
Source: eBioMedicine. 2022 Jun 1;81:104082. doi: 10.1016/j.ebiom.2022.104082 (PMC9155985; doi:10.1016/j.ebiom.2022.104082)

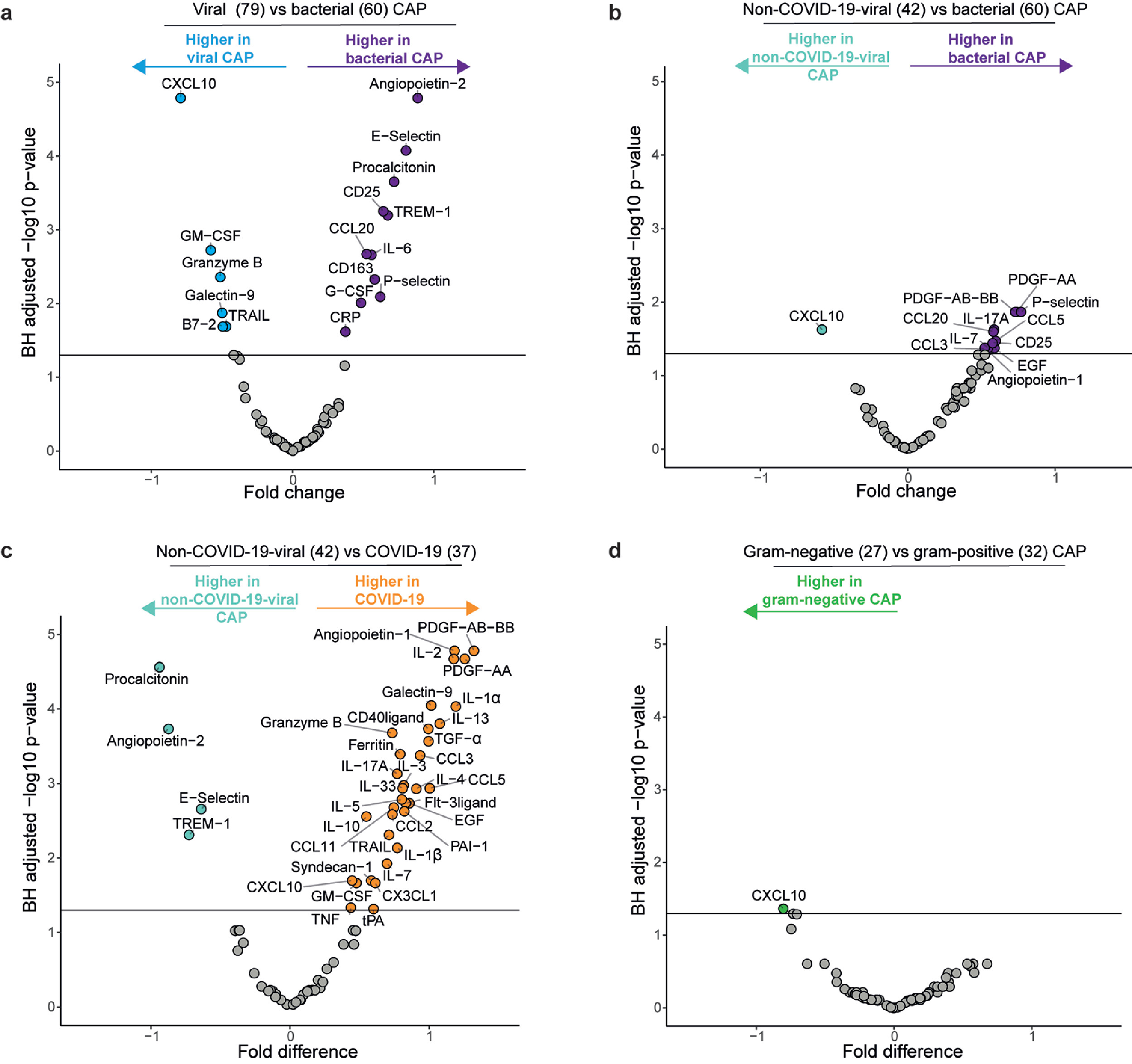

Supplement: Supplementary file 6 [file mmc6.jpg]
